# Factors associated with adverse pregnancy outcome and psychological outcomes during re-pregnancy: a cross-sectional study

**DOI:** 10.3389/fpsyg.2026.1834444

**Published:** 2026-07-22

**Authors:** Ling Liu, Chongyu Yue, Yan Wang, Yiqian Wang, Meng Zhang

**Affiliations:** Department of Obstetrics, The Affiliated Hospital of Qingdao University, Qingdao, China

**Keywords:** adverse pregnancy outcomes, psychological outcomes, recurrent spontaneous abortion, re-pregnancy, uncertainty

## Abstract

**Background:**

Recurrent spontaneous abortion (RSA) leads to both huge psychological trauma and adverse pregnancy outcomes. This study aimed to investigate the status of perceived uncertainty during re-pregnancy in patients with RSA and identify the associated factors of uncertainty.

**Methods:**

This cross-sectional study used a convenience sampling method to select pregnant women in the process of re-pregnancy with recurrent spontaneous abortion as subjects. Patients were recruited from a tertiary A-level hospital from June to November 2025. Data were collected using a self-designed general demographic questionnaire, the Intolerance of Uncertainty Scale-12, the Generalized Anxiety Disorder Scale, the Simple Coping Style Questionnaire, and the Perceived Social Support from Family scale. Univariate and multiple linear regression analyses were employed to investigate factors associated with perceived uncertainty during re-pregnancy.

**Results:**

A total of 230 questionnaires were distributed, and 218 valid questionnaires were collected, with a recovery rate of 94.8%. The mean score of intolerance of uncertainty was (36.02 ± 10.76). Multiple linear regression analyses revealed that education level, monthly family income, number of abortions, history of late-term miscarriage, history of assisted reproductive technology, social support from family, generalized anxiety disorder, and copying style were significant independent factors associated with the perception of uncertainty (*p* < 0.05).

**Conclusion:**

The level of perceived uncertainty during re-pregnancy in patients with recurrent spontaneous abortion is at a moderate level. Medical staff should pay attention to the early identification of uncertainty in patients, enhance the psychological support for patients, and improve their mental health.

## 1 Introduction

Recurrent spontaneous abortion (RSA) is a common pregnancy complication with an incidence rate typically ranging from 1 to 5%. It is defined as the occurrence of two or more spontaneous abortions before 24 or 28 weeks of pregnancy (Yao et al., 2024; Zhang et al., 2010). RSA not only leads to huge psychological and spiritual trauma but also results in adverse outcomes of subsequent pregnancy, such as recurrent miscarriage, pre-eclampsia, and preterm delivery, causing economic burden to the individual, family, and medical system (Jia et al., 2024). RSA Patients are at an elevated risk of experiencing recurrent miscarriages upon subsequent pregnancies with a recurrence risk as high as 70–80%. RSA has multifactorial causes, and currently, 40–50% of cases remain unexplained (Christiansen et al., 2005).

When women with RSA become pregnant again, their previous experiences with pregnancy loss result in heightened expectations for the current pregnancy’s outcome, as well as increased concerns about fetal health and the effectiveness of treatment. However, because the causes of RSA can vary widely, treatment protocols differ significantly among medical institutions. This variability makes it challenging to assess treatment effectiveness and predict fetal development accurately. Compared to women without a history of RSA, those with RSA experience face greater uncertainty regarding the unknowns of pregnancy and their own psychological well-being. This uncertainty has been associated with higher levels of distress, anxiety, and poor birth outcomes (Staneva et al., 2015). Excessively high levels of perceived uncertainty may worsen negative emotions in pregnant women, potentially increasing the risk of adverse outcomes such as preterm birth, low birth weight, and pregnancy-related complications (Eleje et al., 2024; Jiang et al., 2025; Kotera et al., 2025). Given the correlation between lower psychological well-being and poor perinatal outcomes, more studies are needed to examine the prevalence of uncertainty in RSA patients during subsequent pregnancies. Identifying the factors associated with uncertainty is essential for developing targeted interventions to help mitigate uncertainty.

The most commonly used tools for measuring uncertainty included the Mishel Uncertainty in Illness Scale (MUIS) (Mishel, 1988) and the Intolerance of Uncertainty Scale (IUS) (Carleton et al., 2007). MUIS primarily measures the cognitive state of individuals dealing with their illness due to a lack of information about their disease, treatment options, and prognosis. In contrast, IUS measures individuals’ cognitive, emotional, and behavioral responses to uncertain situations and future events, reflecting their personality trait of intolerance to uncertainty. For RSA patients who become pregnant again, the greatest challenge they face is not merely the uncertainty of disease information (as measured by the Mishel scale), but also the unknowns regarding the outcome of the future pregnancy, the deep concerns about whether they can successfully carry the baby, and the fears of unexpected events during the entire pregnancy process. This leads to a pervasive sense of negative expectations and overwhelming anxiety about future adverse events. The IUS precisely measures this low tolerance for uncertainty trait.

While the concept of uncertainty in the context of illness has been studied extensively among nurse scholars, research on uncertainty specifically in high-risk pregnant women has been limited. Most studies have focused on the impact of uncertainty on the psychological well-being in pregnant women (Matthias, 2010; Giurgescu et al., 2006; Çevik and Yağmur, 2018). There remains a significant gap in targeted research exploring the factors associated with perceived uncertainty during the re-pregnancy process in RSA patients.

Therefore, this study aims to explore the factors associated with the uncertainty during the re-pregnancy process in RSA patients. Using the health ecological model as its theoretical framework, this research comprehensively considers the interactive influence of individuals and their social environments. It is expected that the findings can provide a foundation for developing targeted interventions to reduce uncertainty, improve psychological outcomes, and increase the success of pregnancy and delivery in RSA patients.

## 2 Methods

### 2.1 Participants

This study adopted a cross-sectional design and employed a convenience sampling method to select patients with RSA during re-pregnancy. The patients were recruited from the reproductive medicine clinics of the affiliated hospital of Qingdao University (a tertiary grade-A general hospital) from June to November 2025. Informed consent was obtained from all participants. Inclusion criteria: ① A history of two or more spontaneous abortions within 28 weeks of gestation, including complete, incomplete, missed, or inevitable abortion, as well as biochemical pregnancy; ② Age 20 or older and currently pregnant; ③ No communication barriers, with the ability to understand the study’s purpose and complete the questionnaire independently; ④ Willing to voluntarily participate and cooperate with the survey. Exclusion criteria: ① Coexisting physical diseases (e.g., malignant tumors) and/or severe psychiatric disorders; ② Severe visual or hearing impairment; ③ Acute phase of other diseases, or severe impairment of liver, kidney, heart, or lung function.

According to the Kendall sample size estimation method, the sample size was set to be 10–15 times the number of variables. This study included a total of 14 variables. Considering a 20% sample loss rate, the sample size was determined to be 175–262 cases, resulting in a final sample size of 230 cases.

### 2.2 Study instruments

#### 2.2.1 General information questionnaire

A self-designed questionnaire was developed to collect patients’ general information, which included two sections: (1) Socio-demographic data: age, marital history, place of residence, educational level, employment status, average monthly household income, healthcare payment method, etc. (2) Disease-related data: number of miscarriages, history of late-term abortion (gestational age at miscarriage between 12 and 28 weeks), history of assisted reproductive technology.

#### 2.2.2 Intolerance of uncertainty scale-12 (IUS-12)

The IUS-12 is a 12-item scale designed to assess negative beliefs about and reactions to uncertainty. It is a simplified version of the original 27-item Intolerance of Uncertainty Scale (IUS-27), initially developed by Freeston et al. (1994). Carleton et al. (2007) created this simplified version to measure individuals’ responses to uncertain situations and events. Zhang et al. (2017) translated and validated the scale for use with Chinese university students in 2017. It consists of two parts, prospective anxiety (7 items) and inhibitory anxiety (5 items). Prospective anxiety represents fear and worry about future uncertain events, while inhibitory anxiety reflects inhibitory behaviors and experiences in the face of uncertainty. Each item is rated on a 5-point Likert scale ranging from 1 (“Not at all characteristic of me”) to 5 (“Entirely characteristic of me”). The total score ranges from 12 to 60, with higher scores indicating greater intolerance of uncertainty. The scale demonstrates good internal consistency, with a Cronbach’s *α* coefficient of 0.878 (Zhang et al., 2017).

#### 2.2.3 Generalized anxiety disorder scale (GAD-7)

The GAD-7 scale was originally developed by Spitzer et al. in 2006 for screening generalized anxiety disorder and evaluating symptom severity (Spitzer et al., 2006). This tool has been widely used as a measurement tool for anxiety symptoms (Shi et al., 2025; Wang et al., 2025; Wu R. Y. et al., 2025; Zhong et al., 2020). In 2010, He et al. translated and validated the scale in a study involving 600 outpatients (He et al., 2010). Consisting of 7 items, the scale assesses the frequency of anxiety symptoms, such as nervousness and excessive worry, experienced over the past two weeks. Each item is rated on a 4-point Likert scale ranging from 0 (“not at all”) to 3 (“nearly every day”), yielding a total score between 0 and 21. Scores are interpreted as follows: <5 indicates minimal anxiety, 5–9 mild anxiety, 10–14 moderate anxiety, and ≥15 severe anxiety. The scale demonstrates good internal consistency, with a Cronbachs *α* coefficient of 0.898, and test–retest reliability of 0.856 (He et al., 2010).

#### 2.2.4 Simplified coping style questionnaire (SCSQ)

The SCSQ was developed by Xie (1998) based on the theory of coping styles, which was designed to measure an individual’s coping styles when facing stress. The 20-item scale consists of two dimensions: Positive Coping (item 1–12) and Negative Coping (item 13–20). Each item is rated on a 4-point Likert scale from 0 (“never use”) to 3 (“often use”). The total score range is 0–60. The higher the SCSQ score, the more the individual tends to adopt a positive or negative coping style. The SCSQ has good reliability and validity. The Cronbach’s *α* coefficients for the two dimensions are 0.89 and 0.78, respectively.

#### 2.2.5 Social support from family

This scale was developed and validated by two American experts, Procidano and Heller. The scale consists of 20 items, and each item is scored 0 or 1, with 0 indicating “no” and 1 indicating “yes.” Some items are reverse-scored. The total score ranges from 0 to 20, and the higher the score, the higher the level of family support. The Cronbach’s α coefficient is 0.815, and the test–retest reliability is 0.893.

### 2.3 Data analysis

Statistical description was conducted using SPSS 27.0 software. Count data were expressed as frequencies and percentages. Measurement data conforming to a normal distribution were expressed as mean ± standard deviation. Univariate and multiple linear regression analysis was conducted to determine the independent associated factors of perceived uncertainty during re-pregnancy. A *p* value of less than 0.05 was considered statistically significant.

## 3 Results

### 3.1 Demographic characristics of the participants

**Figure 1:**
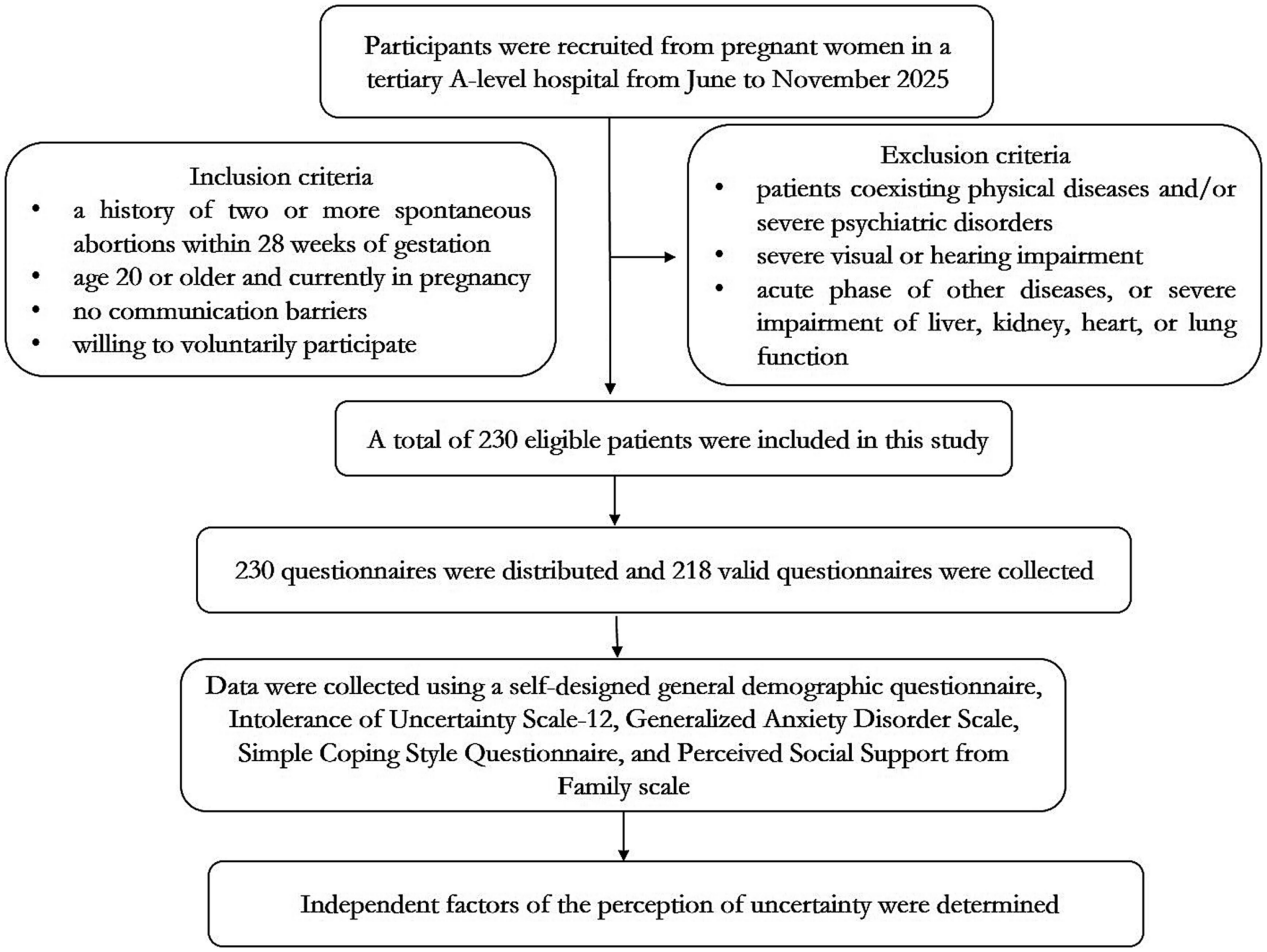
A flowchart of participants.

A flowchart of participants was illustrated in Figure 1. A total of 230 questionnaires were distributed, and 218 valid questionnaires were retrieved, with a valid questionnaire recovery rate of 94.8%. The single-factor analysis revealed that household income, employment, medical insurance, educational level, history of late miscarriage, number of miscarriages, history of assisted reproductive therapy, social support from family, generalized anxiety disorder, and copying style were significant independent factors for perceived uncertainty (*p* < 0.05). The general demographic information and univariate analysis results are shown in Table 1.

**Table 1:** Uncertainty and general demographic information of participants (*n* = 218).

Characteristics	*n* (%)	Score of IUS-12	F/t	*p*
Age (years)			0.912^a^	0.403
20–29	62 (28.4)	34.52 ± 10.92		
30–39	138 (63.3)	36.50 ± 10.62		
≥40	18 (8.3)	37.50 ± 11.41		
Place of residence			−1.868^b^	0.063
Urban	162 (74.3)	35.22 ± 10.22		
Rural	56 (25.7)	38.32 ± 11.99		
Education level			8.151^a^	<0.001
High school or below	33 (15.1)	38.94 ± 8.44		
College or university	144 (66.1)	36.99 ± 11.31		
Post-graduate	41 (18.8)	30.27 ± 8.37		
Monthly family incomes (RMB)			10.264^a^	<0.001
Low (<5,000)	14 (6.4)	39.36 ± 7.65		
Middle (5,000–20,000)	151 (69.3)	37.66 ± 11.48		
High (>20, 000)	53 (24.3)	30.47 ± 6.82		
Employment			−2.525^b^	0.012
Employed	165 (75.7)	34.99 ± 10.45		
Unemployed	53 (24.3)	39.23 ± 11.20		
Medical expense payment method			−2.921^b^	0.004
Medical insurance	170 (78.0)	34.91 ± 10.58		
Self-payment	48 (22.0)	39.96 ± 10.60		
Marriage status			−1.162^b^	0.246
First marriage	203 (93.1)	35.79 ± 10.76		
Remarriage	15 (6.9)	39.13 ± 10.63		
Number of abortions			−2.271^b^	0.024
2	123 (56.4)	34.58 ± 11.44		
≥3	95 (43.6)	37.88 ± 9.56		
History of late-term miscarriage			−2.861^b^	0.005
No	168 (77.1)	34.90 ± 10.90		
Yes	50 (22.9)	39.78 ± 9.45		
History of assisted reproductive technology			−4.502^b^	<0.001
No	155 (71.1)	34.01 ± 10.72		
Yes	63 (28.9)	40.95 ± 9.25		
Lack of knowledge of RSA				
No	160 (73.4)	34.94 ± 10.82	−2.477^b^	0.014
Yes	58 (26.6)	38.98 ± 10.1		
Family support			−4.044	<0.001
Strong	150 (68.8)	34.10 ± 10.87		
Poor	68 (31.2)	40.25 ± 9.29		
Coping style			−2.976^b^	0.003
Positive	166 (76.1)	34.83 ± 10.62		
Negative	52 (23.9)	39.83 ± 10.43		
Generalized anxiety disorder			−3.260^b^	0.001
Low	178 (81.7)	34.92 ± 10.87		
High	40 (18.3)	40.92 ± 8.86		

a F.

b t.

### 3.2 Univariate analysis of perceived uncertainty

The total score of IUS-12 scale for RSA patients during re-pregnancy was 36.02 ± 10.76 points, with 21.25 ± 7.05 points for the anticipatory anxiety and 14.77 ± 5.64 points for the inhibitory anxiety. The univariate analysis indicated statistically significant differences in perceived uncertainty about the following factors: family income, residential, educational level, mode of medical payment, employment, number of abortions, history of late-term miscarriage, history of assisted reproductive technology, social support from family, generalized anxiety disorder, and copying style (*p* < 0.05) (Table 1).

### 3.3 Correlation between perceived uncertainty and anxiety, coping styles

The Generalized Anxiety Disorder Scale (GAD-7) score among the 218 re-pregnant RSA patients was 6.85 ± 3.82 points. Uncertainty was positively correlated with anxiety (r = 0.879, *p* < 0.001). On the Simplified Coping Style Questionnaire, score for the Positive Coping was 22.51 ± 9.25 points, while the Negative Coping was 14.76 ± 6.52 points. Uncertainty showed a negative correlation with positive coping (r = −0.541, *p* < 0.001) and a positive correlation with negative coping (r = 0.145, *p* = 0.017).

### 3.4 Multivariate linear regression analysis of perceived uncertainty

The variables with statistical significance in the univariate analysis were taken as independent variables and the score of the IUS-12 scale for recurrent pregnancy as the dependent variable. A multivariate linear regression analysis was conducted to explore the factors most associated with perceived uncertainty. Coding of independent variables and multiple linear regression results of associated factors of uncertainty are provided in Table 2. The linear regression analysis results showed that monthly household income, history of assisted reproductive technology, anxiety, family support and coping style were the associated factors for the perceived uncertainty during the re-pregnancy process of RSA patients (*p* < 0.05).

**Table 2:** Coding of independent variables and multiple linear regression results of associated factors of uncertainty.

Factors	Assignment	B	SE	β	t	*P*
Constant		−15.562	7.595		−2.049	0.042
Education level	high school = 1, university = 2, postgraduate = 3	−2.317	0.984	−0.125	−2.355	0.019
Monthly household income (yuan)	<5,000 = 1, 5,000–20,000 = 2, > 20,000 = 3	−2.627	1.126	−0.128	−2.334	0.021
Employment	Employed = 0, unemployed = 1	0.414	1.359	0.017	0.305	0.761
Medical expense payment method	Medical insurance = 0, self-payment = 1	2.816	1.358	0.109	2.072	0.040
Number of abortions	2 = 0, ≥3 = 1	6.951	1.453	0.321	4.786	<0.001
History of late-term miscarriage	No = 0, yes = 1	6.516	1.395	0.255	4.670	<0.001
History of assisted reproductive technology	No = 0, yes = 1	5.922	1.376	0.250	4.303	<0.001
Lack of knowledge of RSA	No = 0, yes = 1	5.005	1.389	0.206	3.605	<0.001
Social support from family	Entered as the original values	9.124	1.390	0.394	6.566	<0.001
Negative copying	Entered as the original values	5.198	1.555	0.206	3.343	<0.001
Anxiety	Entered as the original values	3.930	1.465	0.142	2.683	0.008

## 4 Discussion

### 4.1 Status of uncertainty level in RSA patients

The score of the IUS-12 for RSA patients during re-pregnancy indicates a moderate level of perceived uncertainty. Previous studies (Ticconi et al., 2020) have suggested that RSA patients are at a greater risk of pregnancy complications compared to those with normal pregnancies, which may lead to increased psychological burden and increased sensitivity to uncertain outcomes, thereby elevating their perceived uncertainty.

### 4.2 Factors associated with uncertainty in RSA patients during re-pregnancy

#### 4.2.1 Education level and knowledge of RSA

This study found that education level and knowledge of RSA were significant factors for higher uncertainty. RSA patients with high education level experienced lower levels of uncertainty. Lack of information or RSA knowledge was related to higher uncertainty. Patients with limited understanding of disease pathogenesis, recurrence risks and standardized management tended to feel confused about their physical condition and future pregnancy outcomes. Without systematic popularization of RSA-related knowledge, individuals were more likely to generate negative cognitive emotions and persistent ambiguous distress. Improving health education targeted at low-education populations may effectively reduce uncertainty and optimize their psychological status (Mu et al., 2024; Dennehy et al., 2023).

#### 4.2.2 Generalized anxiety disorder

This study found that perceived uncertainty is strongly correlated with anxiety (r = 0.879, *p* < 0.001), which was similar to the findings reported by Huntley et al. (2022). RSA patients undergoing pregnancy reattempt have a higher anxiety level than normal pregnant women. During the re-pregnancy process, due to previous adverse pregnancy experiences, RSA patients will overly focus on their own health conditions and have doubts about fetal development, drug efficacy, and test results. Relevant research (Dugas et al., 2005) has suggested that individuals with a higher uncertainty are prone to negative cognition, which can lead to excessive worry, anxiety, and even symptoms of depression, and an increase in negative emotions and enhanced uncertainty. Nurses can provide perinatal health care knowledge and pregnancy guidance, alleviate their negative emotions such as anxiety, reduce the perceived uncertainty level, and ensure the safety of both mother and baby (Yao et al., 2022).

#### 4.2.3 Coping styles

This study found that higher perceived uncertainty was significantly associated with lower positive coping (r = −0.541, *p* < 0.001) and higher negative coping (r = 0.145, *p* = 0.017) in RSA patients during re-pregnancy. A potential explanation is that many patients were young and employed, with demanding work schedules that sometimes interfered with follow-up visits, possibly reducing positive coping. Inadequate follow-up can limit medical support, which may increase uncertainty, especially as re-pregnant RSA patients often have heightened needs for treatment and monitoring guidance. Furthermore, some patients without a clear diagnosis despite repeated evaluations may perceive medical interventions as ineffective, leading to negative coping, such as discontinuing treatment or questioning care plans, which can further raise uncertainty. Therefore, healthcare providers should monitor the coping strategies, promote emotional and instrumental support to reduce patients’ uncertainty.

#### 4.2.4 Monthly household income

This study revealed that a lower household income was associated with a higher level of perceived uncertainty in re-pregnancy in RSA patients, which was consistent with that of Dennehy et al. (2023). As a complex challenge in reproductive medicine, RSA requires a series of complex and diverse screening tests for etiological clarification, which may increase the financial burden of low-income patients and potentially undermine their trust in medical treatment, thereby increasing their sense of uncertainty. RSA patients with a low income often have limited access to reliable information on reproductive health and face multiple pressures from family members and financial pressure, resulting in a higher level of uncertainty. Thus, establishing a dedicated RSA education section on hospital official platforms could offer patients tailored reproductive health information, further alleviating pregnancy-related anxieties.

#### 4.2.5 History of assisted reproductive technology (ART)

This study showed that RSA patients with a history of ART have a higher perceived uncertainty level during their subsequent pregnancies (*p* = 0.001), which is similar to previous results. This may be because ART for conception is a long-term, multi-stage, and high-risk treatment method. Patients’ unfamiliarity with the treatment protocols, combined with uncertainties regarding therapeutic outcomes, contributes to a higher perceived uncertainty level during subsequent pregnancy attempts in RSA patients with a history of ART. Studies indicate that ART patients typically exhibit greater information needs, stronger perceptions of uncertainty, and a higher likelihood of experiencing negative emotions throughout treatment. Nursing staff should therefore provide health education and psychological support to ART patients to provide regular full-cycle health guidance, improve their emotional well-being and reduce the perceived uncertainty level.

#### 4.2.6 Perceived social support from family (PSS-Fa)

The social support from family is a significant factor for uncertainty in RSA Patients during re-pregnancy. Pregnant women with strong social support from family reported lower uncertainty, as emotional comfort and practical assistance from relatives help ease their fear of recurrent fetal loss. Without consistent care and understanding from family members, affected women tend to ruminate on adverse pregnancy outcomes alone, which greatly intensifies their ambiguous psychological distress (Howlett et al., 2021; Yu et al., 2025; Wu Y. et al., 2025).

### 4.3 Implications

This study has significant theoretical and practical implications. Theoretically, it enriches the application of the uncertainty management theory and health ecology model in the context of RSA re-pregnancy, expanding the understanding of how individual, contextual, and psychological factors interact to shape perceived uncertainty in high-risk pregnancy settings. Practically, the identification of key associated factors provides insights for clinical psychologists, obstetric nurses, and healthcare providers to develop targeted psychological interventions, alleviate perceived uncertainty and improve the mental health of RSA patients during re-pregnancy. For patients exhibiting higher levels of uncertainty, clear and targeted disease-related information, structured health education, and supportive care should be provided. Nurses must explore and seek to understand the individual concerns and factors contributing to uncertainty during Re-pregnancy in patients with RSA. Understanding individual concerns and the factors contributing to uncertainty enables nurses to offer clinical and emotional support, as well as tailored coping strategies that can help reduce uncertainty during re-pregnancy in RSA patients.

### 4.4 Strengths and limitations

This study has several significant strengths that enhance its academic value and contribution to the field of psychology. This study directly addresses perceived uncertainty, a core psychological distress factor which is closely associated with perinatal mental health outcomes but lacks systematic exploration in this specific group. Second, the study adopts a systematic and rigorous methodological design. It comprehensively identifies associated factors of perceived uncertainty, including sociodemographic characteristics, pregnancy-related factors, and psychological factors. The use of validated scales, such as the Intolerance of Uncertainty Scale-12, the Generalized Anxiety Disorder Scale, the Simple Coping Style Questionnaire, and the Perceived Social Support from Family scale, ensures the reliability and validity of data collection, meeting the methodological standards for empirical research. However, this study has several limitations. First, this study employs a convenience sample from one hospital. This may limit the generalizability of findings to all RSA patients and the findings may not be representative of the entire population of pregnant women in China. Another limitation is that this study only uses a cross-sectional survey method and fails to dynamically track the changes in perceived uncertainty. Further research can further explore the trajectory and characteristics of the perceived uncertainty level in RSA patients during re-pregnancy, and provide a theoretical basis for formulating targeted interventions.

## 5 Conclusion

In conclusion, the perceived uncertainty during the re-pregnancy process in RSA patients is at a moderate level. The perceived uncertainty is associated with factors such as education level, monthly family income, number of abortions, history of late-term miscarriage, history of assisted reproductive technology, social support from family, generalized anxiety disorder, and copying style. This approach can assist patients in adopting positive coping strategies, reducing their sense of uncertainty, and ultimately improving their psychological well-being.

## Data Availability

The raw data supporting the conclusions of this article will be made available by the authors, without undue reservation.
